# Wnt7a Inhibits IL-1β Induced Catabolic Gene Expression and Prevents Articular Cartilage Damage in Experimental Osteoarthritis

**DOI:** 10.1038/srep41823

**Published:** 2017-02-06

**Authors:** Averi L. Gibson, Carrie K. Hui Mingalone, Andrea T. Foote, Tomoya Uchimura, Ming Zhang, Li Zeng

**Affiliations:** 1 Program in Cellular, Molecular, and Developmental Biology, Sackler School of Graduate Biomedical Sciences, Tufts University, Boston, MA 02111, USA; 2 Department of Integrative Physiology and Pathobiology, Tufts University School of Medicine, Boston, MA 02111, USA; 3 Department of Rheumatology, Tufts Medical Center, 800 Washington Street, Boston, MA 02111, USA; 4 Department of Orthopedics, Tufts Medical Center, 800 Washington Street, Boston, MA 02111, USA

## Abstract

Wnt7a is a protein that plays a critical role in skeletal development. However, its effect on cartilage homeostasis under pathological conditions is not known. In this study, we found a unique inverse correlation between Wnt7a gene expression and that of MMP and IL-1β in individual human OA cartilage specimens. Upon ectopic expression in primary human articular chondrocytes, Wnt7a inhibited IL-1β-induced MMP and iNOS gene expression. Western blot analysis indicated that Wnt7a induced both canonical Wnt signaling and NFAT and Akt non-canonical signaling. Interestingly, inhibiting the canonical and Akt pathway did not affect Wnt7a activity. However, inhibiting the NFAT pathway impaired Wnt7a’s ability to inhibit MMP expression, suggesting that Wnt7a requires NFAT signaling to exert this function. *In vivo*, intraarticular injection of lentiviral Wnt7a strongly attenuated articular cartilage damage induced by destabilization of the medial meniscus (DMM) OA-inducing surgery in mice. Consistently, Wnt7a also inhibited the progressive increase of joint MMP activity in DMM animals. These results indicate that Wnt7a signaling inhibits inflammatory stimuli-induced catabolic gene expression in human articular chondrocytes and is sufficient to attenuate MMP activities and promote joint cartilage integrity in mouse experimental OA, demonstrating a novel effect of Wnt7a on regulating OA pathogenesis.

Osteoarthritis (OA) is a common joint disease that causes debilitating chronic pain worldwide^1^. OA is characterized by progressive joint destruction, including loss of the articular cartilage, chondrocyte cell death, and ectopic bone formation (i.e. osteophytes)^2^,^3^. Wear-and-tear of cartilage from repeated use, general aging and ligament injury plays a critical role in OA pathogenesis^4^,^5^. A key player in OA development is inflammation^6^. Higher levels of pro-inflammatory cytokines such as IL-1β cause cartilage damage by increasing the expression of catabolic matrix enzymes such as matrix metalloproteinases (MMPs), which ultimately leads to cartilage matrix degradation^6^.

The Wnt pathway is a highly conserved signaling pathway that plays a crucial role in development and disease^7^,^8^. Wnt molecules are known to activate two types of pathways: the canonical and non-canonical pathways^9^. The canonical pathway involves the accumulation of β-catenin in the nucleus and induction of downstream target genes, typically Axin2^10^. The non-canonical pathways include multiple signaling pathways, such as calcium and NFAT signaling, the planar cell polarity pathway, and Akt activation^11^,^12^,^13^. The canonical and non-canonical pathways may also antagonize each other. For example, the calcium signaling pathway has been shown to stimulate β-catenin degradation and inhibit the canonical pathway^9^,^14^.

Human genetic studies identified the Wnt inhibitor sFRP3 as a potential regulator of OA susceptibility, which spurred investigation into the role of Wnt signaling in OA^15^,^16^. Indeed, many Wnt proteins are expressed in healthy cartilage^10^ and several studies have shown changes in Wnt signaling with OA development in both human and animal studies^17^,^18^,^19^. However, studies on downstream Wnt signaling components in OA progression present controversial results. For example, while ablation of β-catenin *in vivo* resulted in degeneration of articular cartilage^20^,^21^, overexpression of β-catenin *in vivo* also caused an OA-like phenotype^18^. In addition, overexpression of a canonical Wnt pathway inhibitor DKK-1 and antisense knockdown of DKK-1 both inhibited OA articular cartilage damage^22^,^23^. Similarly inconclusive results have also been seen related to calcium signaling^24^,^25^,^26^,^27^. Perhaps such seemingly contradictory data indicate that an intricate balance in Wnt signaling must be maintained for optimal cartilage homeostasis. Such a balance is likely to be delicately maintained by multiple Wnt molecules. However, despite the studies of downstream Wnt signaling components, no Wnt ligands have been tested in an *in vivo* experimental OA setting.

*In vitro* experiments showed that Wnt3a, Wnt5a, Wnt7a, and Wnt7b induced MMP expression in rodent chondrocytes under normal, non-pathological conditions^28^,^29^,^30^,^31^,^32^. But additional studies also indicated that Wnt3a decreased MMP expression under IL-1β treatment and normal conditions in human chondrocytes^33^. Thus, it is important to investigate the effect of Wnts in both human and rodent systems, and under normal and pathological conditions.

Wnt7a acts through both the canonical and non-canonical Wnt pathways in a cell type-dependent manner^12^,^34^,^35^. While it has been shown to be expressed in articular cartilage^10^, the effect of Wnt7a in OA or human chondrocytes under pathological conditions has not been examined. In this study, we investigated the expression of Wnt7a in human OA cartilage and found an intriguing non-linear inverse correlation with the expression of several catabolic genes. We further examined the effect of Wnt7a ectopic expression in human primary articular chondrocytes under inflammatory conditions, as well as in the destabilization of the medial meniscus (DMM) surgery OA mouse model. Our results indicate a beneficial effect of Wnt7a on chondrocytes *in vitro* and *in vivo*, and suggest that NFAT signaling is necessary for this effect of Wnt7a.

## Results

### Wnt7a is downregulated in human OA cartilage and inversely correlated with catabolic gene expression

**Figure 1:**
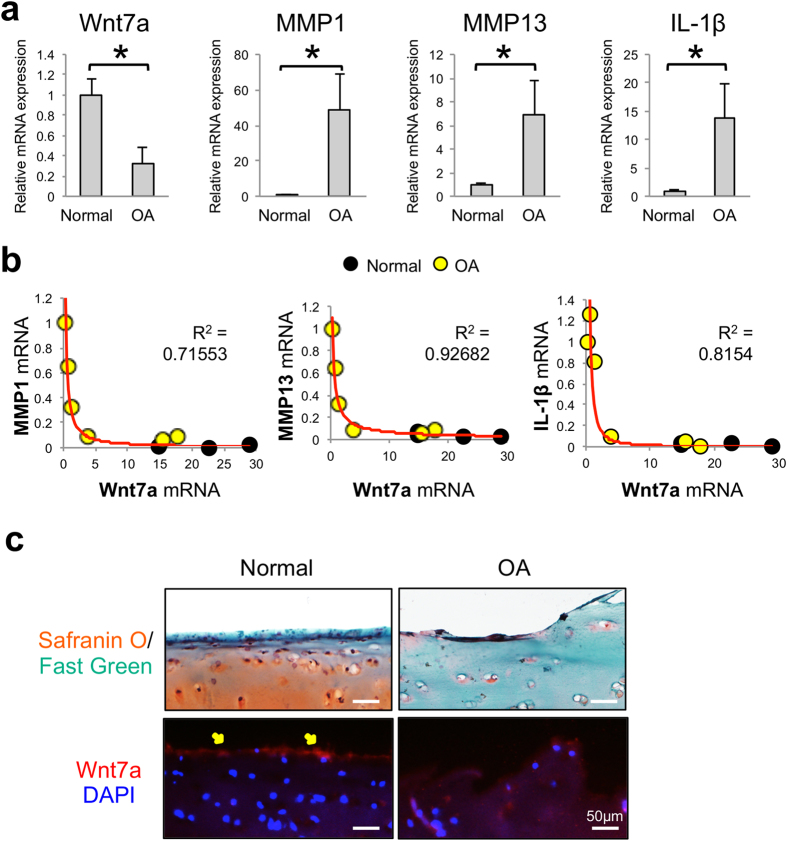
Wnt7a mRNA expression is reduced in human OA and inversely correlated with catabolic gene expression. **(a**) RT-PCR analysis of Wnt7a and MMP1, MMP3 and IL-1β gene expression in tibial cartilage specimens isolated from normal cadaveric and OA patients. The age and sex of human cartilage specimens used were: normal (n = 4): 84/M, 75/M, 65/F, and 49/F. OA specimens (n = 6): 53/F, 63/F, 65/F, 72/M, 73/F, 73/M. (**b**) Correlation analysis of Wnt7a mRNA expression with catabolic gene expression in normal cadaveric and OA patient samples. Data points from the six OA samples scatter toward the left of the graphs (yellow dots), and those from the four normal samples are scattered to the right of the graphs (black dots), with two of the normal sample points overlapping with one another. (**c**) Normal and OA human cartilage samples stained with safranin O/fast green and an anti-Wnt7a antibody. DAPI was used to visualize the nuclei. Diffuse staining was observed in both normal and OA cartilage, but strong Wnt7a protein expression was detected on the articular surface (arrows) in normal cartilage. Experiments were performed in triplicates with technical duplicates. A student’s t-test was used for evaluating the statistical significance of normal vs. OA gene expression. All correlation analyses were performed using a Spearman correlation and were statistically significant (p < 0.05). Data are shown as mean ± SEM. *p < 0.05.

To establish the relevancy of Wnt7a in OA, we first assayed for Wnt7a gene expression in human OA cartilage samples from joint replacement surgeries and non-OA cadaveric controls. In a prior study using these specimens, our lab demonstrated higher levels of catabolic genes and lower levels of anabolic genes were present in the OA samples^36^. Here, we found that Wnt7a was significantly reduced in the human OA samples compared to healthy controls, thus exhibiting an opposite trend from MMP1, MMP13, and IL-1β (Fig. 1a). When the expression of Wnt7a was compared to that of catabolic genes in each sample, including both the normal and OA cartilage specimens, a striking inverse correlation emerged (Fig. 1b). We found that higher expression of Wnt7a trended with lower expression of MMP1, MMP13, and IL-1β. Vice versa, lower expression of Wnt7a paired with higher expression of MMP1, MMP13, and IL-1β. Interestingly, this correlation was not linear. Apparently, when Wnt7a expression surpassed a certain level, it was correlated with a dramatic decrease in the catabolic gene expression, and these samples tended to be the normal cartilage specimens with little cartilage damage. When Wnt7a expression fell below this level, it was correlated with a dramatic increase in catabolic gene expression, and these samples tended to be the OA specimens with high levels of cartilage damage (Fig. 1b). Immunohistological (IHC) analysis was also performed to evaluate Wnt7a protein levels in human articular cartilage. We found that sections of normal cartilage showed a stronger Wnt7a fluorescence signal on the articular surface than OA cartilage (Fig. 1c), suggesting that Wnt7a protein levels were also decreased in OA. As our cartilage specimens tended to represent two ends of the spectrum in joint health (normal vs. severe OA), we did not correlate gene expression and protein levels with OA severity, which would require more specimens with early to moderate OA.

### Wnt7a inhibits IL-1β induced catabolic gene expression in human articular chondrocytes *in vitro*

**Figure 2:**
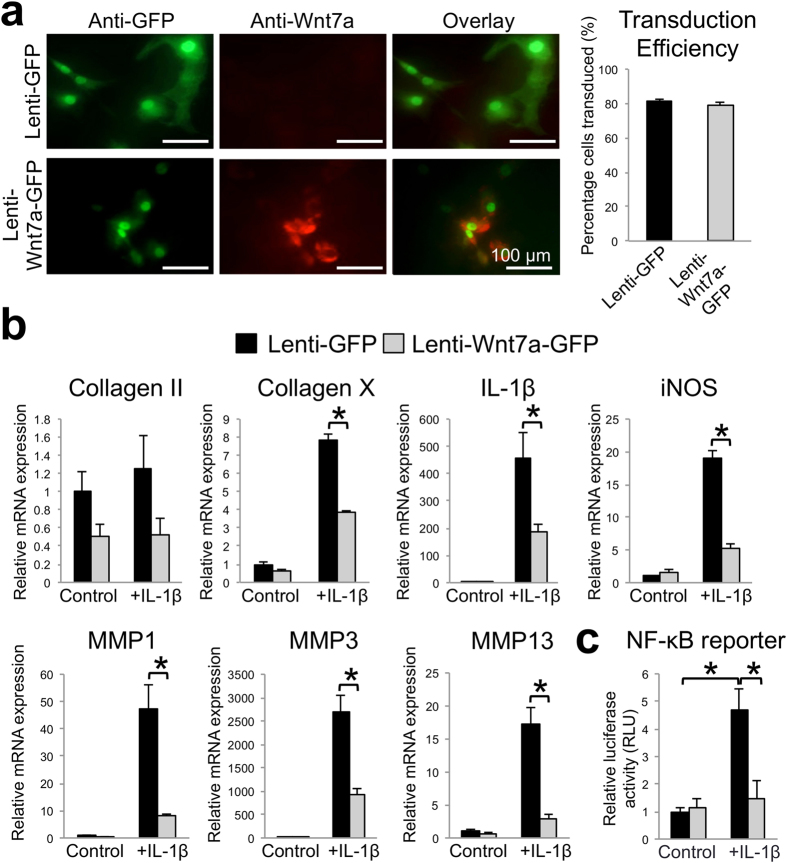
Wnt7a ectopic expression reduces IL-1β induced upregulation of OA-related genes and NF-κB activity *in vitro*. (**a**) Representative images of Wnt7a IF analysis of normal human articular chondrocytes (nHACs) after infection with lenti-GFP or lenti-Wnt7a-GFP. Wnt7a immunofluorescence images were overlaid with GFP expression. Percentage of cells positive for lenti-GFP or lenti-Wnt7a-GFP was quantified based on GFP production, which were 81.4% and 78.8%, respectively. (**b**) RT-PCR analysis of nHACs gene expression after infection with lenti-GFP or lenti-Wnt7a-GFP with or without IL-1β (5 ng/mL). Lenti-Wnt7a-GFP viral infection significantly reduced IL-1β upregulation of Collagen X, IL-1β, iNOS, and several MMPs. A student’s t-test was used for evaluating the statistical significance between the gene expression of lenti-GFP and lenti-Wnt7a cells under either control or IL-1β conditions. (**c)** NF-κB luciferase assay of NF-κB activity after infection with lenti-GFP or lenti-Wnt7a-GFP and treatment with or without IL-1β (5 ng/mL). Relative luciferase activity in reference to Renilla luciferase internal control is shown. Experiments were conducted with biological triplicates and repeated at least three times. Analysis of variance (ANOVA) with post-hoc tests was used for evaluating the statistical significance of the GFP vs. Wnt7a and control vs. IL-1β luciferase activity. All data are shown as mean ± SEM. *p < 0.05.

Because of the inverse correlation between Wnt7a and the catabolic genes MMP13, MMP1, and IL-1β in human cartilage, we directly tested the role of Wnt7a in an established *in vitro* system where the pro-inflammatory cytokine IL-1β is used to induce chondrocyte catabolic gene expression^33^,^36^,^37^. Wnt7a was introduced to cultured normal primary human articular chondrocytes (nHACs) by viral expression. A commercially available lentivirus encoding human Wnt7a with a separate GFP module (lenti-Wnt7a-GFP) was used, and a lentivirus encoding GFP alone (lenti-GFP) served as a control. We first confirmed that these viruses resulted in Wnt7a and GFP ectopic expression in chondrocytes *in vitro* at equivalent rates (Fig. 2a). We then analyzed gene expression upon Wnt7a and IL-1β treatment. IL-1β induced the expression of multiple MMPs, iNOS, Collagen X, and IL-1β itself. Significantly, Wnt7a ectopic expression led to a dramatic inhibition of the IL-1β-induced upregulation of all these genes (Fig. 2b). There was no significant effect on collagen II mRNA expression by IL-1β or Wnt7a.

To investigate the mechanism by which Wnt7a inhibited IL-1β induction of these genes, we examined NF-κB activation in these samples by evaluating an NF-κB luciferase reporter, as NF-κB is a key mediator of IL-1β activity^38^,^39^,^40^. As expected, IL-1β activated the NF-κB reporter (Fig. 2c)^33^,^36^. However, treatment with the Wnt7a lentivirus inhibited IL-1β-induced NF-κB reporter activity, indicating that Wnt7a inhibited NF-κB activity.

### Wnt7a inhibits joint cartilage destruction *in vivo*

To test whether Wnt7a can inhibit joint damage *in vivo*, we utilized the “destabilization of the medial meniscus” (DMM) model, which is a well established joint-injury OA mouse model^41^,^42^. We first evaluated Wnt7a mRNA expression in the DMM cartilage vs the sham joint and found that Wnt7a was decreased upon DMM surgery (Supplementary Fig. 1), which is consistent with our observation using human OA cartilage specimens. Furthermore, we also observed that treatment with lentiviral Wnt7a (i.e. lenti-Wnt7a-GFP) inhibited IL-1β-induced MMPs and iNOS in murine chondrocytes (Supplementary Fig. 1), which is also consistent with our data using human articular chondrocytes.

**Figure 3:**
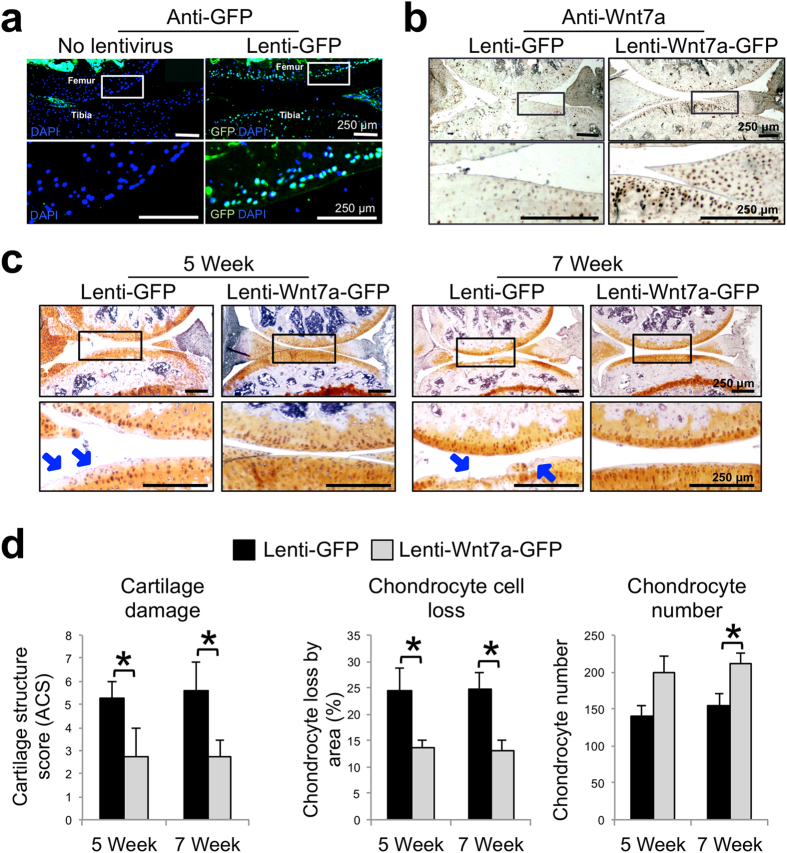
Wnt7a ectopic expression prevents cartilage damage in the DMM OA model *in vivo*. (**a**) Representative images of GFP immunofluorescence (IF) analysis of mouse knee joints 5 weeks post DMM surgery with or without lenti-GFP joint infection. Rectangles denote areas shown in higher magnification. Both the superficial and deeper zones of the articular cartilage were infected. (**b**) Representative images of Wnt7a IF analysis in mouse knees injected with lenti-GFP or lenti-Wnt7a-GFP at 5 weeks post DMM surgery. Rectangles denote areas shown in higher magnification. Wnt7a IF confirmed ectopic expression of Wnt7a. Quantification analysis based on GFP and Wnt7a expression from at least four independent images for each treatment showed the percentage of tibial articular chondrocytes positive for lenti-GFP or lenti-Wnt7a-GFP was 55.1% and 57.7% respectively. (**c**) Representative images showing safranin O/hematoxylin stained joint sections at 5 or 7 weeks post DMM surgery in mouse knees injected with lenti-GFP or lenti-Wnt7a-GFP. Rectangles denote areas shown in higher magnification. Arrows denote areas of articular cartilage damage. Lenti-GFP control knees showed significantly more cartilage damage compared to knees ectopically expressing Wnt7a. (**d**) Cartilage structure scoring (ACS), chondrocyte loss and chondrocyte numbers in the tibia were quantified at 5 or 7 weeks post DMM in mouse knees injected with lenti-GFP or lenti-Wnt7a-GFP. n = 6 mice/group with 9 sections/joint used for blind scoring. Nonparametric statistical analyses were used for evaluating the statistical significance of the histological scoring systems at each time point. Data are shown as mean ± SEM. *p < 0.05.

Having observed consistent effects of Wnt7a on human and mouse chondrocytes *in vitro*, we evaluated the effect of Wnt7a *in vivo* by intraarticularly injecting lenti-Wnt7a-GFP or lenti-GFP at 1 and 2 weeks post DMM surgery. When their expression was evaluated at five weeks post surgery, we found that lenti-GFP had infected chondrocytes in the superficial and deeper zones of the articular cartilage, as well in the meniscus (Fig. 3a). No evidence of viral infection of the subchondral bone was seen. Additionally, Wnt7a IHC indicated that while endogenous Wnt7a is present in the articular cartilage and meniscus, lenti-Wnt7a-GFP treatment resulted in significantly higher Wnt7a protein expression in the joint cartilage (Fig. 3b).

Safranin O staining revealed that articular cartilage damage was evident in lenti-GFP-infected joints at both 5 and 7 weeks post surgery. Strikingly, the damage was greatly diminished in lenti-Wnt7a-GFP infected knees (Fig. 3c). This was further confirmed by scoring using the articular cartilage structure (ACS) score for quantification of cartilage damage (Fig. 3d). Our histological analysis also revealed that the Wnt7a knee joints had a significant reduction in chondrocyte cell loss, and subsequently a higher chondrocyte number, compared to GFP controls^43^,^44^ (Fig. 3d). These results strongly indicate that Wnt7a treatment after the onset of OA can preserve articular cartilage against cartilage matrix damage and cell loss.

**Figure 4:**
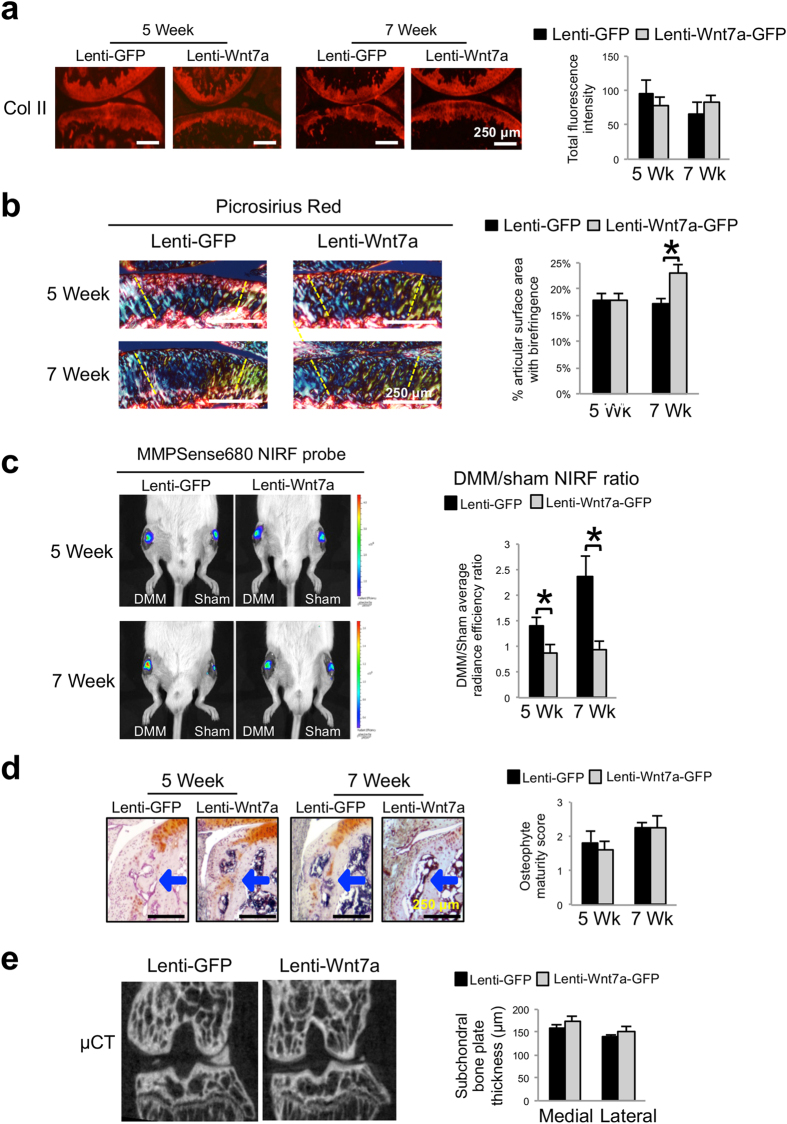
Wnt7a reduces joint MMP activity during OA development, but has no effect on subchondral bone. (**a**) Representative images of Collagen II IHC analysis 5 or 7 weeks post DMM surgery in mouse knees injected with lenti-GFP or lenti-Wnt7a-GFP. Quantification analysis based on total Collagen II fluorescence of the femur and tibia from at least 4 sections/treatment indicated there were no significant differences among groups. (**b**) Representative images and semi-quantitative analysis of picrosirius red staining showing collagen fiber thickness and organization of articular cartilage from knee joints at 5 or 7 weeks post DMM surgery, as viewed under polarized light. Areas used for subsequent quantification of birefringence signals are delineated by dashed lines. (**c**) Representative *in vivo* near infrared fluorescence (NIRF) images taken two hours after 4 μL intraarticular injection of MMPSense680 in each knee. Each lenti-GFP and lenti-Wnt7a-GFP-infected mouse was imaged serially at 5 and 7 weeks post surgery. The average radiance efficiency emitted from each knee was quantified from the collected NIRF images and the DMM average radiance efficiency was divided by the sham average radiance efficiency to internally calibrate each signal. Quantification analysis demonstrated that mice ectopically expressing Wnt7a in the joint have reduced NIRF signals compared to lenti-GFP controls. (**d**) Representative histological images of osteophyte development with safraninO/hematoxylin staining and scoring of osteophyte maturity at 5 or 7 weeks post DMM surgery in mouse knees injected with lenti-GFP or lenti-Wnt7a-GFP. Arrows indicate osteophytes. Osteophyte maturity was quantified. (**e**) Representative micro-CT cross-sections of the knee joint and quantification of medial and lateral tibial subchondral bone plate thickness at 7 weeks post DMM surgery in mouse knees injected with lenti-GFP or lenti-Wnt7a-GFP. Micro-CT quantification demonstrated no differences between the lenti-GFP and lenti-Wnt7a-GFP groups. n = 7 mice/group for NIRF imaging and 6 mice/group for microCT imaging and histology. A student’s t-test was used for evaluating the statistical significance between GFP and Wnt7a mice at each individual time point. All data are shown as mean ± SEM. *p < 0.05.

We next proceeded to evaluate collagen II, the principal collagen in cartilage, by IHC. Quantification of the fluorescence signals showed no significant differences among the treatment groups (Fig. 4a). This is not unexpected as collagen proteins have a long half-life^45^ and may not be degraded to the point of being completely absent in our experimental period, which may be why significant changes in collagen II protein expression at early time points in the DMM model are rarely reported. However, collagen fiber structure and conformation may be altered before frank reduction in collagen II protein levels becomes visible^46^. To determine whether collagen II structure was altered, we examined collagen fibers under polarized light after picrosirius red staining. It has been well established that the picrosirius dye can bind to collagen and strongly enhances collagen’s birefringent property. Under polarized light, collagen fibers exhibit a variety of colors, with thicker collagen fibers exhibiting colors of higher wavelengths^47^,^48^. We found mouse articular cartilage showed collagen fibers with yellow and green colors, while the subchondral bone contained collagen fibers with red colors (Fig. 4b). This is consistent with the fact that cartilage consists of collagen II fibers and bone consists of collagen I fibers, and that collagen I fibers are much thicker than collagen II fibers^49^. Since cartilage structural alteration is most pronounced in the central region^50^,^51^,^52^, the amount of birefringence signals in this region was quantified. We did not observe a difference between lenti-Wnt7a treated joint with the lenti-GFP control at 5 weeks post-surgery, but did find a higher level of birefringent signal in lenti-Wnt7a treated joint at 7 weeks post-surgery compared to GFP control samples (Fig. 4b), which is suggestive of higher levels of collagen fibers at 7 weeks. It would be interesting to quantitatively determine collagen fiber thickness and orientation in these samples and to determine whether these changes were caused by alteration of collagen degradation and fiber synthesis.

As MMP activities have been shown to be upregulated in OA^53^,^54^, we investigated whether treatment with Wnt7a affected the activities of MMPs in the joint by near infrared fluorescence (NIRF) live imaging. For NIRF imaging, we used the fluorescent probe MMPSense680, which contains a consensus-MMP cleavage site and is thus activated by multiple MMPs, including MMP13, MMP9 and MMP3^42^,^55^. In the presence of active MMPs, cleavage of this probe causes the emission of fluorescence that falls into the near infrared range^56^,^57^. We have previously shown that the DMM/sham fluorescence emission ratio of MMPSense680 reflects the trajectory of joint destruction in the DMM OA model^42^. Here, when we analyzed the images from joints injected with MMPSense680, we found that the DMM/sham signal ratio was significantly reduced in the lenti-Wnt7a-GFP treated joints at 5 weeks compared to the lenti-GFP controls (Fig. 4c). At 7 weeks, while the DMM/sham signal ratio in lenti-GFP control knee joints had increased, lenti-Wnt7a-GFP treated joints remained low (Fig. 4c). Therefore, this result suggests that Wnt7a ectopic expression in the joint reduced MMP activities *in vivo*. Even though collagen II IHC did not reveal any significant differences between the GFP and Wnt7a-treated groups at these time points, it is possible that the reduction in MMP activity by Wnt7a will reduce collagen fiber denaturing or disorganization and eventually result in appreciable differences in collagen that are detectable by IHC at later time points.

Since features of OA include osteophyte formation and subchondral bone sclerosis^44^,^58^, we also assessed osteophyte maturity by histological analysis and subchondral bone by microCT (Fig. 4d–e). However, these analyses did not yield any significant differences between the GFP and Wnt7a treated groups, which could be due to the fact that no Wnt7a ectopic expression was observed in these areas.

### Wnt7a activates both canonical and non-canonical pathways in chondrocytes

**Figure 5:**
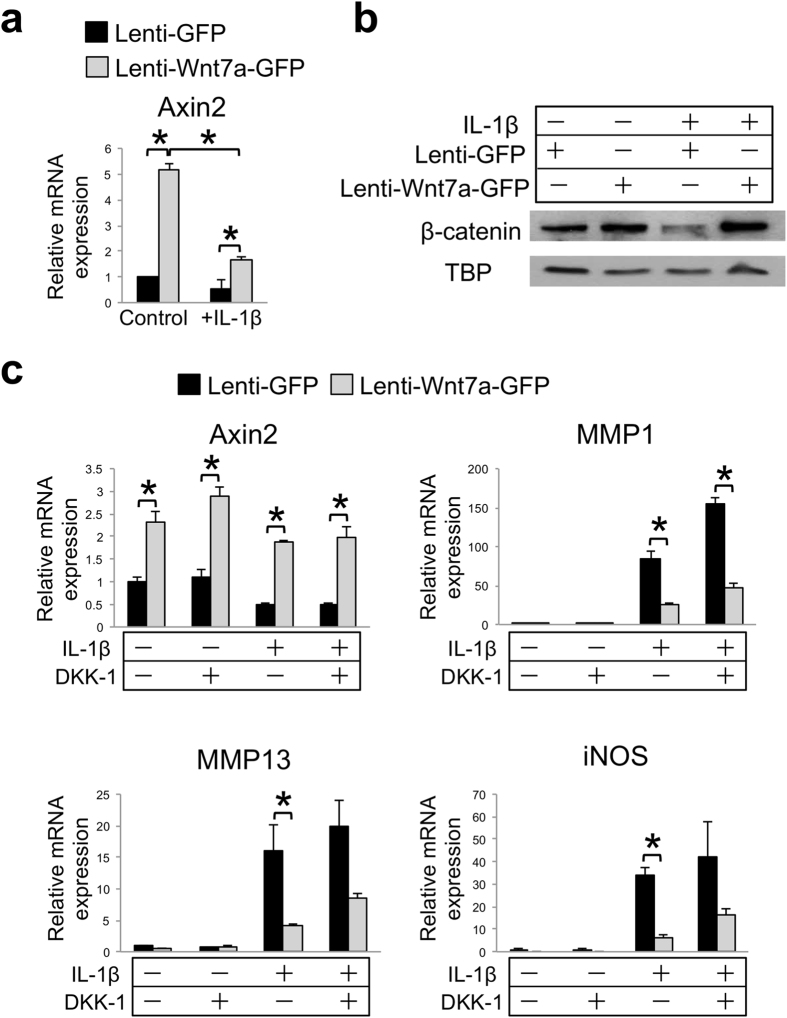
Inhibition of canonical Wnt signaling does not diminish Wnt7a’s inhibition of IL-1β activity in human chondrocytes. (**a**) RT-PCR analysis of Axin2 mRNA expression in nHACs after infection with lenti-GFP or lenti-Wnt7a-GFP, and cultured with or without IL-1β (5 ng/mL). (**b**) Western blot analysis of nuclear β-catenin in nHACs after infection with lenti-GFP or lenti-Wnt7a-GFP in the presence or absence of IL-1β (5 ng/mL). TATA-binding protein (TBP) served as a loading control. Original films for cropped images can be found in the supplementary information file. (**c**) RT-PCR analysis of Axin2, MMP1, MMP13 and iNOS gene expression after treatment with DKK-1 (250 ng/mL), on nHACs infected with lenti-GFP or lenti-Wnt7a-GFP, and cultured with or without IL-1β (5 ng/mL). Each experiment had three biological repeats/treatment, and at least three independent experiments were performed. A student’s t-test was used for evaluating the statistical significance between the gene expression of lenti-GFP and lenti-Wnt7a cells under each individual experimental condition. All data are shown as mean ± SEM. *p < 0.05.

Next, we investigated the biochemical signaling pathways by which Wnt7a may be exerting its effects. Since Wnt7a has been shown to activate multiple signaling pathways, markers of these pathways were evaluated upon lenti-Wnt7a-GFP infection of human articular chondrocytes (nHACs). We first evaluated Axin2 induction and β-catenin nuclear localization, which are key events in the Wnt canonical pathway^7^. We found that Axin2 mRNA expression and β-catenin nuclear localization were both induced by Wnt7a (Fig. 5a,b). To determine if canonical pathway signaling mediates the activity of Wnt7a, we added the canonical Wnt pathway inhibitor DKK-1, which specifically binds LRP6^59^. Our result demonstrated that DKK-1 treatment at 250 ng/mL did not block Wnt7a-induced Axin2 expression (Fig. 5c), even though DKK-1 at the same concentration was able to inhibit Wnt3a-induced Axin2 expression in the same cells (Supplementary Fig. 2)^14^,^33^. High dose treatment with DKK-1 at 500 ng/mL also did not have an effect on the Wnt7a upregulation of Axin2 (Supplementary Fig. 2). Furthermore, a dominant-negative Tcf4 did not block the Wnt7a upregulation of Axin2 expression either (Supplementary Fig. 2), suggesting that Wnt7a induced Axin2 using an alternative mechanism. Accordingly, we found that DKK-1 treatment did not have an effect on Wnt7a inhibition of IL-1β-induced MMP1, although it did cause an additional increase in IL-1β-induced MMP1 level (Fig. 5c). A similar trend was observed for MMP13 and iNOS expression analysis (Fig. 5c). On the other hand, DKK-1 treatment did enhance overall MMP1 expression under IL-1β treatment (Fig. 5c). These results suggest that canonical signaling may be inhibitory towards IL-1β, but it is not essential for Wnt7a-mediated inhibition of catabolic gene expression.

**Figure 6:**
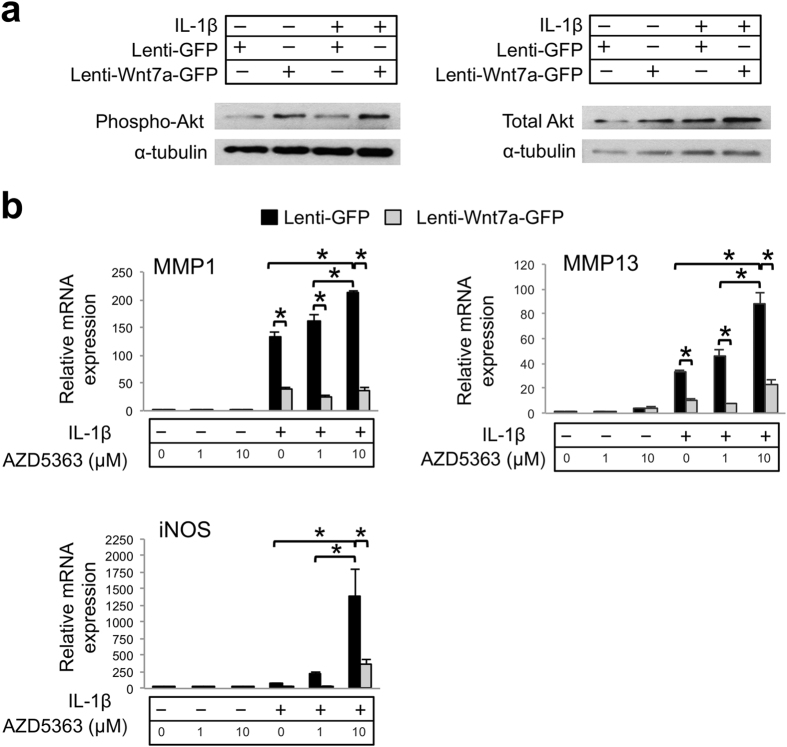
Inhibition of Akt signaling does not diminish Wnt7a’s inhibition of IL-1β activity in human chondrocytes. (**a**) Western blot analysis of Akt protein expression after nHACs were infected with lenti-GFP or lenti-Wnt7a-GFP and cultured with or without IL-1β (5 ng/mL). Results demonstrated that phosphorylated Akt was upregulated with Wnt7a ectopic expression compared to lenti-GFP controls. Original films for cropped images can be found in the supplementary information file. **(b**) RT-PCR analysis of MMP1, MMP13 and iNOS gene expression after treatment with Akt inhibitor AZD5363 (1 or 10 uM) on nHACs infected with lenti-GFP or lenti-Wnt7a-GFP and cultured in the presence of absence of IL-1β (5 ng/mL). Each experiment had three biological repeats/treatment, and at least three independent experiments were performed. Analysis of variance (ANOVA) with post-hoc tests was used for evaluating the statistical significance between the gene expression of lenti-GFP and lenti-Wnt7a cells across all of experimental conditions. All data are shown as mean ± SEM. *p < 0.05.

We then tested the non-canonical pathways. Because Wnt7a has been shown to activate the Akt pathway in muscle and rabbit chondrocytes^12^,^60^, we evaluated Akt phosphorylation by performing Western blot analysis. Our data indicated that lenti-Wnt7a-GFP treatment led to Akt phosphorylation in articular chondrocytes as well (Fig. 6a). To determine whether Wnt7a inhibits IL-1β through the Akt pathway, we used a well-established Akt inhibitor AZD5363^61^,^62^. Similar to the case of DKK-1, administering the Akt inhibitor significantly increased MMP1, MMP13 and iNOS expression induced by IL-1β, but it did not block Wnt7a’s ability to inhibit IL-1β’s activity (Fig. 6b). The reason why inhibiting Akt led to increased MMP expression in the GFP control cells is not clear. Since iNOS mRNA was also strongly induced by the Akt inhibitor, and nitric oxide has been reported to sustain NFkB activation and induce MMPs^63^,^64^, it is possible that iNOS induction plays a role in the MMP induction in this process. Nevertheless, even though our data suggests that Akt signaling may be inhibitory to IL-1β, it also indicates that it is not required for Wnt7a’s ability to inhibit IL-1β induction of MMPs, despite being activated by Wnt7a.

**Figure 7:**
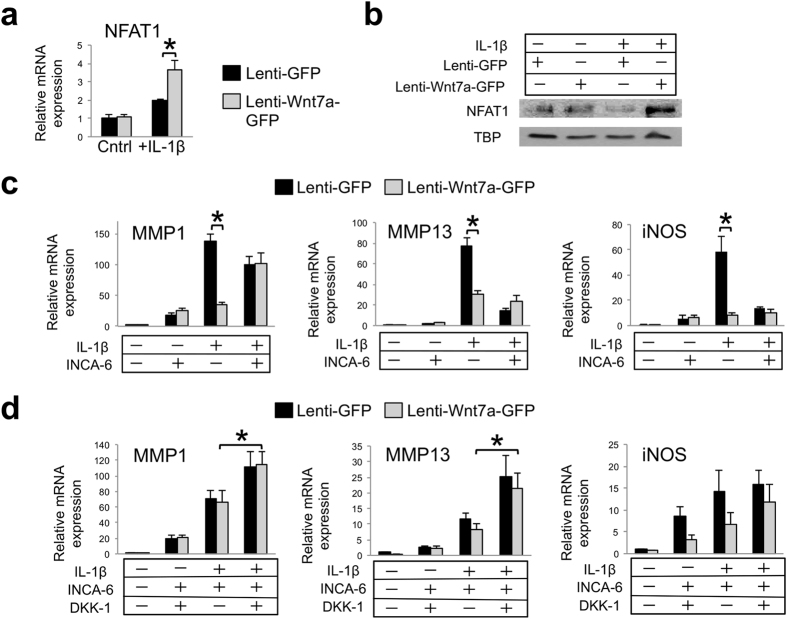
Inhibition of NFAT signaling attenuates the effect of Wnt7a on MMP inhibition in human chondrocytes. (**a**) RT-PCR analysis of NFAT1 gene expression in nHACs after infection with lenti-GFP or lenti-Wnt7a-GFP, and cultured with or without IL-1β (5 ng/mL). NFAT1 expression was upregulated with Wnt7a ectopic expression in the IL-1β group only. (**b)** Western blot analysis of nuclear NFAT1 protein expression after nHACs were infected with lenti-GFP or lenti-Wnt7a-GFP and cultured with or without IL-1β (5 ng/mL). TBP served as a loading control. Original films for cropped images can be found in the supplementary information file. (**c)** RT-PCR analysis of MMP1, MMP13 and iNOS after treatment with 20 μM of INCA-6 on nHACs infected with lenti-GFP or lenti-Wnt7a-GFP and treated with or without 5 ng/mL IL-1β for 2 days. (**d**) RT-PCR analysis of MMP1, MMP13 and iNOS gene expression after treatment with 20 μM of INCA-6 and/or 250 ng/mL DKK-1 on nHACs infected with lenti-GFP or lenti-Wnt7a-GFP and cultured in the presence of absence of IL-1β (5 ng/mL). Each experiment had three biological repeats/treatment, and at least three independent experiments were performed. Analysis of variance (ANOVA) with post-hoc tests was used for evaluating the statistical significance between the gene expression of lenti-GFP and lenti-Wnt7a cells across all of experimental conditions. All data are shown as mean ± SEM. *p < 0.05.

NFAT signaling is another noted component of Wnt non-canonical signaling, as it mediates the calcium pathway^13^. We found that Wnt7a induced NFAT1 mRNA expression and NFAT nuclear localization (Fig. 7a,b). Interestingly, the addition of IL-1β further augmented NFAT1 gene expression, which may involve crosstalk from the downstream pathways of IL-1β and Wnt7a^65^,^66^,^67^,^68^,^69^.

To determine if NFAT signaling was necessary for Wnt7a action, we applied a well established NFAT inhibitor INCA-6, which we confirmed to have the ability to inhibit an NFAT luciferase reporter (Supplementary Fig. 3). Significantly, we found that while lenti-Wnt7a-GFP inhibited IL-1β-induced MMP1 expression, it was not able to do so when INCA-6 was added (Fig. 7c). In the presence of INCA-6, IL-1β-induced MMP13 and iNOS expression was much reduced, but Wnt7a did not further inhibit IL-1β-induced MMP13 expression (Fig. 7c). Therefore, administration of INCA-6 abolished the differences in MMP1, MMP13 and iNOS expression between the lenti-Wnt7a-GFP and lenti-GFP groups under IL-1β treatment (Fig. 7c), suggesting that Wnt7a’s inhibition of IL-1β in chondrocytes requires NFAT signaling. It is worth noting that INCA-6 by itself increased basal levels of MMP1 and iNOS expression in the absence of IL-1β, but inhibited their expression in the presence of IL-1β (Fig. 7). It is unclear why this is the case, but perhaps a moderate level of NFAT signaling is optimal for chondrocytes.

Since the canonical Wnt signaling and non-canonical Wnt signaling pathways are known to crosstalk, and Wnt7a induced both pathways, we applied both the NFAT inhibitor INCA-6 and the canonical Wnt pathway inhibitor DKK-1 to the chondrocytes. Strikingly, combinatorial treatment of INCA-6 and DKK-1 resulted in an additional increase in MMP and iNOS expression compared with INCA-6 treatment alone (Fig. 7d). Therefore, while inhibiting the canonical Wnt pathway alone was not sufficient to significantly block Wnt7a’s inhibition of IL-1β-induced catabolic gene expression, inhibiting both the canonical pathway and the non-canonical pathway did, suggesting that NFAT signaling and Wnt canonical signaling act synergistically to mediate the activity of Wnt7a in chondrocytes under inflammatory conditions.

## Discussion

Human Wnt7a deficiency results in severe birth defects with limb patterning abnormalities^70^. However, whether Wnt7a affects articular cartilage maintenance in OA conditions in humans is not known. We have shown a striking non-linear inverse correlation of Wnt7a mRNA expression and the catabolic genes MMP1, MMP13, and IL-1β in human cartilage samples, which propelled us to investigate the effect of Wnt7a treatment on chondrocytes under pathological conditions. Our study constitutes the first study to investigate the effect of Wnt7a on chondrocytes under pathological conditions *in vivo* and *in vitro*. Importantly, ectopic expression of Wnt7a *in vivo* began at one week post surgery; therefore, Wnt7a protected articular cartilage against OA-related damage even when OA development had already begun, suggesting our result is promising from a therapeutic standpoint.

It is intriguing that while Wnt7a could activate both canonical and non-canonical Wnt pathways in chondrocytes, as been found in other cell types^12^,^34^,^35^, the canonical Wnt pathway alone was not required for Wnt7a’s striking inhibition of IL-1β-induced MMP expression. Rather, the canonical Wnt7 pathway cooperated with the non-canonical Wnt pathway to mediate the activity of Wnt7a in inhibiting IL-1β-induced catabolic gene expression. In a prior study, Wnt7a was found to promote chondrogenic dedifferentiation in rabbit chondrocytes through the canonical pathway, but enhanced chondrocyte survival through phosphatidylinositol 3-kinase and Akt^60^. Wnt3a has also been demonstrated in chondrocytes could activate both the canonical and non-canonical signaling pathways, which regulated cell proliferation and cell differentiation respectively. However, these studies were performed exclusively under normal, non-pathological conditions^14^.

Under inflammatory cytokine treatment, Ma *et al*. showed that Wnt3a and Wnt7b inhibited MMP expression in human chondrocytes specifically through the canonical Wnt pathway; on the other hand, Wnt3a induced MMP expression in mouse chondrocytes^33^. In contrast, we have found consistent effect of Wnt7a in human and mouse chondrocytes. Meanwhile, Ge *et al*. showed that Wnt5a promoted IL-1β-induced MMP expression through activating JNK in rabbit fibrocartilage cells^30^. Therefore, the activity of Wnts apparently depends on whether a normal or pathological condition was used and whether mouse or human chondrocytes were used. This also highlights the importance of investigating individual Wnt ligands, as studies focusing only on the downstream signaling components may not capture this level of complexity. As the canonical Wnt pathway and the non-canonical Wnt pathway have been mostly viewed to be antagonistic of each other in chondrocytes^71^, our study suggests a new notion of how downstream Wnt pathways mediate Wnt activity. Future studies will involve the evaluation of an additional non-canonical Wnt pathway, the planar polarity pathway and JNK signaling, and the investigation of whether the Akt pathway also crosstalk with these other pathways.

Despite the *in vitro* studies using pro-inflammatory cytokines and the knowledge that inflammation is an important component in OA pathogenesis^39^,^72^, it is critical to evaluate the effect of Wnt treatment on OA progression *in vivo*, which is much more complex. None of the above-discussed Wnt molecules have been shown to regulate OA progression *in vivo*. The only experiments involving introducing Wnts into the joint were performed under normal conditions.

In the report by Van Den Bosch *et al*., adenoviral injection of Wnt8a and Wnt16 into the normal joint resulted in OA-like damage to the articular cartilage. As this activity could be blocked by DKK-1, these Wnts acted through the canonical Wnt pathway^73^. However, because both DKK-1 overexpression and anti-sense knockdown reduced joint damage in experimental OA^22^,^23^, whether the canonical Wnt pathway has a similar effect in control as in OA conditions is still unclear. The only non-canonical Wnt that has been tested *in vivo* under normal conditions in articular cartilage is Wnt5a, although Wnt5a did not affect articular cartilage integrity^73^. In our study, we also did not observe a significant difference when lentiviral Wnt7a was injected into the normal joint. However, in the DMM joints, we observed dramatic improvement in cartilage structure and chondrocyte survival, again indicating a difference between testing under control and under OA conditions. During our evaluation, even though both safranin O analysis and NIRF imaging both revealed a beneficial effect from lentiviral Wnt7a treatment, only NIRF imaging was able to detect an increase in the OA phenotype from 5 to 7 weeks post DMM in the lenti-GFP-treated joint. This is consistent with our prior study that indicated that NIRF imaging was more sensitive than the traditional histological methods in evaluating the trajectory of joint destruction over time^42^. Since Wnt7a treatment prevented in the increase in NIRF signal from 5 to 7 weeks post DMM surgery, it suggests that Wnt7a inhibits the *progression* of experimental OA. This study thus also validates the NIRF imaging technology in serving as a sensitive evaluation tool for monitoring OA.

It is not clear how Wnt7a inhibits matrix loss and chondrocyte survival in OA animals. Based on our *in vitro* study, future experiments would involve the investigation of whether the Wnt canonical and/or non-canonical pathways, especially NFAT signaling, mediate Wnt7a’s activity *in vivo*. While NFAT itself has not been directly studied under OA conditions, it was shown to be beneficial to the joint under normal conditions, since the loss of NFAT1 resulted in the spontaneous development of OA-like joint damage in mice^24^. Additionally, NFAT1 and NFAT2 double knockout also caused articular cartilage damage^25^. Furthermore, overexpression of NFAT1 increased collagen gene expression and decreased IL-1β and TNFα gene expression in chondrocytes from aged mice^74^. It has been previously demonstrated that during chondrogenetic differentiation in the mouse limb bud, NFAT signaling can be activated by Wnt5a, which in turn reduced NF-κB activity^75^. Since Wnt7a also induced β-catenin nuclear localization, it will be interesting to test whether the physical interaction of β-catenin and p65 contributed to Wnt7a’s activity in our setting. It is also worth noting that inflammation is only one aspect of OA development *in vivo*. Other factors, such as mechanical stress, also cause cell loss and matrix damage and contribute to OA, especially in the DMM joint destabilization model^76^,^77^,^78^. Thus, it is also possible that Wnt7a inhibits OA by modulating these other aspects.

In summary, here we demonstrate that Wnt7a is a molecule whose expression is reduced in OA cartilage and inversely correlated with catabolic genes. Our *in vitro* and *in vivo* studies have provided unique mechanisms and new perspectives on how Wnt signaling affects cartilage destruction and OA development, and will help identify novel potential therapeutics for OA.

## Methods

### Normal and OA Human Cartilage Tissue Samples

All experiments related to human specimens were performed in accordance with relevant guidelines and regulations. Normal cartilage samples were purchased from the National Disease Research Interchange (NDRI) and Articular Engineering from tibial plateaus of cadaveric joints of subjects with no history or radiographic signs of OA (n = 4, age/sex: 84/M, 75/M, 65/F, and 49/F). No donor identities were provided for these samples. OA cartilage samples were obtained from tibial plateaus of patients undergoing total knee replacement surgery for OA at Tufts Medical Center (n = 6, age/sex: 53/F, 63/F, 65/F, 72/M, 73/F, 73/M). NDRI also provided cartilage samples of both OA and normal cartilage from one donor (85 M). The MANKIN scores for the normal samples are: 84/M = 1, 75/F = 2, 65/F = 3, 85/M = 2; OA samples: 53/F = 6, 63/F = 7, 65/F = 9, 73/F = 7, 73/M = 8, 85 M = 8. All OA cartilage samples were de-identified before we received them and would have otherwise been discarded. Sample collection protocols were reviewed by the Institutional Review Board (IRB) at Tufts University and classified as exempt. Cartilage samples were characterized as previously described^36^.

### Experimental Animals and surgical model of OA

All animal care and experimental procedures were approved by the Institutional Animal Care and Use Committee (IACUC) at Tufts University (Protocol #B2014–11) and performed in accordance with the Animal Welfare Act and the Public Health Service Policy on Humane Care and Use of Laboratory Animals. Wild-type CD1 male mice were purchased from Charles River Laboratories. All mice were caged in groups under standard conditions. Destabilization of the medial meniscus (DMM) surgery was performed on 7 week old CD1 male mice according to the established protocol^41^,^42^. Briefly, under isoflurane anesthesia, the right knee joint was opened along the medial border of the patellar ligament and the medial meniscotibial ligament (MMTL) was severed. Sham surgery, where the MMTL was visualized but not severed, was performed on the left knee of the same mice as an internal control.

### *In Vivo* Lentiviral Treatment

On days 7 and 14 post DMM surgery, 5 μL of lentiviral human Wnt7a-GFP or lentiviral GFP (Open Biosystems) with a titer of 5 × 10^7^ IFU was directly injected through the patellar ligament into the mouse knee joint cavity using a 30 G needle. Both knee joints of each mouse were injected with the same lentivirus. Mice were then euthanized at 5 or 7 weeks post DMM surgery. Viral efficiency of both GFP and Wnt7a viruses was assessed by quantifying the percentage of articular chondrocytes infected by each virus respectively, as visualized by IHC detected protein expression.

### *In Vitro* Human Chondrocyte Cultures

Primary normal human articular chondrocytes (nHACs, Lonza) were redifferentiated in alginate beads for 3 weeks in chondrogenic differentiation media (Lonza) before being used^36^. For viral infection, redifferentiated nHACs were infected with lentiviral GFP (lenti-GFP, control) or lentiviral human Wnt7a-GFP (lenti-Wnt7a-GFP, Open Biosystems) with a titer of 5 × 10^7^ IFU for two days before additional treatments were applied. Viral infection efficiency of both GFP and Wnt7a viruses was assessed by quantifying the percentage of cells infected by each virus respectively, as visualized by GFP signal. For IL-1β treatment, 0 or 5 ng/mL IL-1β (Peprotech) was applied for 4 days prior to RT-PCR analysis, or for 1 hour prior to Western blot analysis. For all signal inhibition experiments, compounds were added to the media for two days. The concentrations of signal inhibitors were: recombinant human DKK-1 (R&D Systems), 250 or 500 ng/mL; Akt inhibitor AZD5363 (Selleckchem), 1 or 10 μM; INCA-6 (Tocris), 20 μM of in 0.2% DMSO. For Tcf4 inhibition, lenti-dnTcf4 (Addgene, plasmid #24310) was used to perform a simultaneous double transfection with the lenti-Wnt7a-GFP and lenti-GFP for two days before IL-1β was added. For experiments involved Wnt3a conditioned medium, L cells stably expressing Wnt3a (ATCC CRL-2647) and L cell parental line (ATCC CRL-2648, control) were used.

### *In Vitro* Murine Chondrocyte Cultures

Primary murine chondrocytes were harvested from the knee joint cartilage of 6-day old mice as described^79^ and expanded in DMEM (Gibco) with 10% FBS, 1% penicillin/streptomycin, and 2% glutamine. They were infected with lenti-Wnt7a-GFP or lenti-GFP (Open Biosystems) with a titer of 5 × 10^7^ IFU for two days and then treated for 0 or 5 ng/mL IL-1β (Peprotech) for an additional 2 days before RT-PCR analysis.

### RNA isolation and real-time RT-PCR

Total RNA from human chondrocytes or mouse knee joints was isolated with the Qiagen RNeasy Mini Kit (Qiagen) and cDNA was generated using M-MLV Reverse Transcriptase (Invitrogen). PCR was performed using the iQ5 Real-Time PCR Detection System (Bio-Rad). RT-PCR analyses of human OA samples were normalized to TATA biding protein (TBP) expression. *In vitro* human and murine chondrocyte cultures were normalized to GAPDH expression^80^. Primer sequences are available upon request.

### Western blots

Cells were lysed with RIPA buffer. Nuclear and cytoplasmic fractions were isolated using NE-PER Nuclear and Cytoplasmic extraction reagents (Pierce). Protein concentrations were determined using the DC^TM^ Protein Assay (Bio-Rad). Following SDS-PAGE, Western Blot analysis was performed using the following primary antibodies: β-catenin (Millipore), NFAT1 (Cell Signaling), pan-Akt (Cell Signaling), phospho-Akt (Ser473, Cell Signaling), TATA-binding protein (TBP, Abcam), and α-tubulin (Developmental Studies Hybridoma Bank). A horseradish peroxidase-conjugated antibody (Millipore) was used as a secondary antibody, and Pierece ECL Western Detection kit (ThermoFisher) was used for protein visualization.

### Luciferase Assay

For the NF-κB luciferase assay, three days after lentiviral infection, chondroyctes were co-transfected with a six-copy NF-κB element-driven luciferase reporter construct (Dr. Gail Sonenshein, Tufts University). Twenty-four hours after transfection, cells were treated with 0 or 5 ng/mL IL-1β (Peprotech) for 16 hours. For the NFAT luciferase assay, 293 T cells were transfected with an NFAT luciferase reporter construct (Dr. Chi-Wing Chow, Albert Einstein College of Medicine). Twenty-four hours after transfection, the cells were treated with 0 or 2 μM ionomycin (Sigma) (as a positive control for inducing the NFAT luciferase reporter) and 0, 4, or 20 μM INCA-6 (Tocris) for 16 hours. For both experiments, a Renilla luciferase construct was co-transfected as an internal control, and relative luciferase units (RLU) were calculated based on the measurement using the Dual-Glo Luciferase Assay System (Promega) and a 1450 MicroBeta Trilux Luminescence Counter (PerkinElmer).

### Histological Analysis

Human cartilage and mouse knee joints were fixed in 4% paraformaldehyde and decalcified in 10% EDTA. Human cartilage specimens were cryosectioned and mouse joints were paraffin sectioned, both at 5 μm thickness. 0.1% safranin O was used for matrix evaluation, and fast green or hematoxylin was used as counterstains. For human OA cartilage samples, OA damage was assessed through Mankin scoring of the Safranin O histology^81^. For mouse joint analysis, Safranin O histology of sham surgery samples was also analyzed to confirm that none of the groups had increased joint damage at baseline. For each sample, 9 stained sections were pooled, randomized and blindly scored for cartilage damage using the established Articular Cartilage Structure (ACS) scoring system^43^. The femur and tibia were scored separately and added together to calculate a total joint score for each section. The sections were blindly analyzed for three additional OA histological parameters, tibial chondrocyte cell loss, tibial chondrocyte cell number, and osteophyte maturity, according to established methods^43^,^44^,^82^. For histological joint analysis, 6 mice/group were used for a total of 24 mice.

### Immunohistochemistry

For IHC analysis of Wnt7a and GFP proteins, heat-induced antigen retrieval in 10 mM citric acid buffer was performed. For Collagen II staining, enzymatic antigen retrieval was done using 0.3% hyaluronidase and 0.15% trypsin (Invitrogen) at 37 °C. Sections were incubated with the following primary antibodies overnight at 4 °C: Wnt7a (Santa Cruz), GFP (Abcam), Collagen II (Abcam); and the following secondary antibodies for 2hrs: Wnt7a staining–donkey anti-goat IgG (H + L) secondary antibody Texas Red conjugate (Jackson) or a biotin-conjugated secondary antibody (Vector) for chromogenic staining; Collagen II staining - goat anti-rabbit IgG (H + L) secondary antibody, Alexa Fluor^®^ 594 conjugate (Invitrogen); GFP - goat anti-chicken IgY (H + L) secondary antibody, Alexa Fluor^®^ 488 conjugate (Invitrogen). Fluorescent sections were counterstained with DAPI. The Vectastain Elite ABC Kit (Vector) was used for chromogenic staining. Sections incubated without the primary antibody served as negative controls for each round of staining.

### Light microscopy

Bright field and fluorescent images were taken using an Olympus IX-71 microscope and Olympus DP70 and DP80 digital cameras. The optical parameters and camera exposure time were kept constant between samples of the same experiment.

### Quantification of collagen II protein levels and fiber content

For quantification of the fluorescent signals from collagen II IHC, and articular cartilage areas were delineated using Photoshop. Then, areas with chondrocytes (lacunae) were eliminated using a Matlab imaging software. Multiple areas outside the articular cartilage were used as background readings. Total fluorescence of all images based on areas of fluorescence and fluorescence intensity was determined using the same Matlab imaging software, and total fluorescence relative to that from 5 week GFP samples was plotted. For visualizing collagen fibers, sections were stained with 0.1% sirius red diluted in saturated picric acid (i.e. picrosirius red staining) and birefringence was observed under polarized light. Areas of articular cartilage were delineated using Photoshop. Since cartilage structural alteration is most pronounced in the central region^50^,^51^,^52^, the amount of birefringence signals in the central tibial articular cartilage was analyzed using a Matlab software. This area encompasses the articular cartilage not covered by the meniscus and accounts for 20% of the articular cartilage surface.

### Near infrared fluorescence (NIRF) *in vivo* imaging and data analysis

For *in vivo* NIRF imaging, 7 mice/group were imaged at 5 and 7 weeks post surgery. To begin, the hair of the mouse hind limb was removed to reduce autofluorescence. Immediately prior to probe injection, the mice were anesthetized and imaged using the IVIS-200 Imaging System (PerkinElmer) with the Cy5.5 filter set (excitation: 615–665 nm, emission: 695–770) for a background reading. Then, under isoflurane anesthesia, 4 μL of 13.3 nmol MMPSense680 (PerkinElmer) was directly injected through the patellar ligament into the knee joint cavity using a 30 G needle. The mice were then allowed unrestricted cage activity for two hours before imaging. For imaging, mice were again anesthetized with isoflurane and an approximately 1.5 cm skin incision was created directly over the patellar ligament of each knee to allow imaging without skin overlying the joint. Images were again acquired using the IVIS-200 Imaging System with the Cy5.5 filter set (excitation: 615–665 nm, emission: 695–770 nm)^42^.

Fluorescence signal intensity was measured using the Living Image software (PerkinElmer). A region of interest (ROI) was drawn over each knee joint to quantify the average fluorescent signal intensity, measured in radiance efficiency, collected over each knee joint in the raw image. The size and shape of the region of interest was kept constant for all knees analyzed. The background fluorescence signal intensity of each mouse knee was measured from the background image taken immediately prior to probe injection and subtracted from the raw data for each knee joint. The measured average radiance efficiency of the DMM knee was divided by the measured average radiance efficiency of the sham knee to internally calibrate each image.

### Micro-Computed Tomography

For micro-CT analysis, 6 mice/group were scanned using the Skyscan 1176 micro-CT scanner (Bruker) at 7 weeks post DMM. The x-ray source was set to a voxel size of 9 μM at 50 kV and 200 μA. A beam filtration filter of 0.5 mm aluminum filter was used and data was recorded every 1° for a total of 180°. Images slices were reconstructed using NRecon software (Bruker) and the 3D data analysis was performed using CTAnalyzer software (Bruker). The epiphysis of the tibia was manually chosen as the region of interest for 3D analysis of the subchondral bone. For subchondral trabeculae, the following parameters were calculated: trabecular bone volume fraction (BV/TV), representing the ratio of trabecular bone volume (BV) to endocortical tissue volume (TV), subchondral bone tissue volume, trabecular thickness, and trabecular separation. The volume of the mineralized osteophytes was measured separately using the same method. Thickness of the medial and lateral subchondral bone plates was determined using the same algorithm used for analyzing trabecular thickness.

### Statistical Analysis

Data are shown as mean ± SEM. The semiquantitative histological scoring systems were evaluated using nonparametric statistical analyses. All other experiments were evaluated with a student’s t-test or analysis of variance (ANOVA) with post-hoc tests for pairwise comparisons. All student’s t-tests were performed as two-tailed analyses. Spearman correlation was used for correlation analyses. Outliers were only removed when statistical significance was acquired using the Grubb’s test. A p value of less than 0.05 was considered significant in all cases.

## Additional Information

**How to cite this article:** Gibson, A. L. *et al*. Wnt7a Inhibits IL-1β Induced Catabolic Gene Expression and Prevents Articular Cartilage Damage in Experimental Osteoarthritis. *Sci. Rep.* **7**, 41823; doi: 10.1038/srep41823 (2017).

**Publisher's note:** Springer Nature remains neutral with regard to jurisdictional claims in published maps and institutional affiliations.

## Supplementary Material

Supplementary Figures
