# Supplementary material for: Wnt7a Inhibits IL-1β Induced Catabolic Gene Expression and Prevents Articular Cartilage Damage in Experimental Osteoarthritis
Source: Sci Rep. 2017 Feb 6;7:41823. doi: 10.1038/srep41823 (PMC5292965; doi:10.1038/srep41823)
Supplement: Supplementary Figures [file srep41823-s1.pdf]

Wnt7a Inhibits IL-1β Induced Catabolic Gene Expression and Prevents Articular Cartilage Damage in Experimental Osteoarthritis

Averi L. Gibson[1,2], Carrie K. Hui Mingalone[1,2], Andrea T. Foote[2], Tomoya Uchimura[1,2], Ming Zhang[3], and Li Zeng[1,2,4,*]

[1] Program in Cellular, Molecular, and Developmental Biology, Sackler School of Graduate Biomedical Sciences, Tufts University, Boston, MA 02111.

[2] Department of Integrative Physiology and Pathobiology, Tufts University School of Medicine, Boston, MA 02111.

[3] Department of Rheumatology, Tufts Medical Center, 800 Washington Street, Boston, MA 02111, USA.

[4] Department of Orthopedics, Tufts Medical Center, 800 Washington Street, Boston, MA 02111, USA.

* Corresponding Author: Li Zeng, Ph.D.

Email: Li.Zeng@tufts.edu

Telephone: (617) 636-2107 Fax: (617) 636-3676

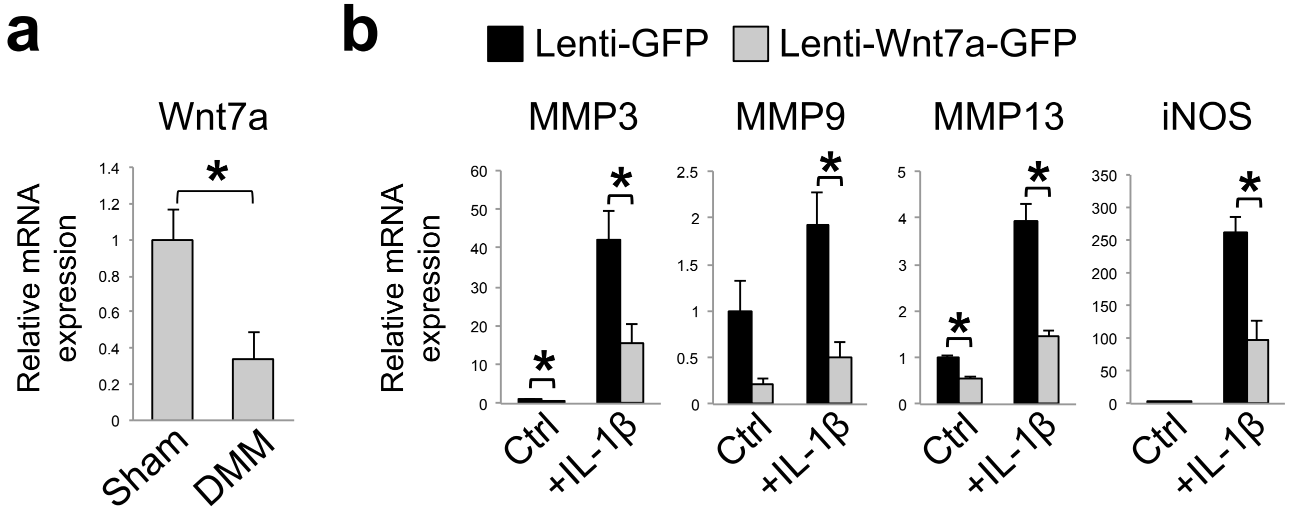

Supplementary Figure 1. Wnt7a ectopic expression reduces IL-1β induced upregulation of OA-related genes in murine chondrocytes *in vitro*. a. RT-PCR analysis of Wnt7a gene expression in tibial cartilage specimens isolated from mouse knees 8 weeks post DMM or sham surgery. A reduced Wnt7a gene expression was observed in the DMM knees compared to sham controls. n = 3 mice/group. **b.** RT-PCR analysis of MMP3, MMP9, MMP13 and iNOS gene expression in murine chondrocytes infected with lenti-GFP or lenti-Wnt7a-GFP and cultured with or without IL-1β (5ng/mL). A student's t-test was used for evaluating the statistical significance of sham vs. DMM gene expression and lenti-GFP vs. lenti-Wnt7a cell gene expression under either control or IL-1β conditions. All data are shown as mean ± SEM. * = $p < 0.05$.

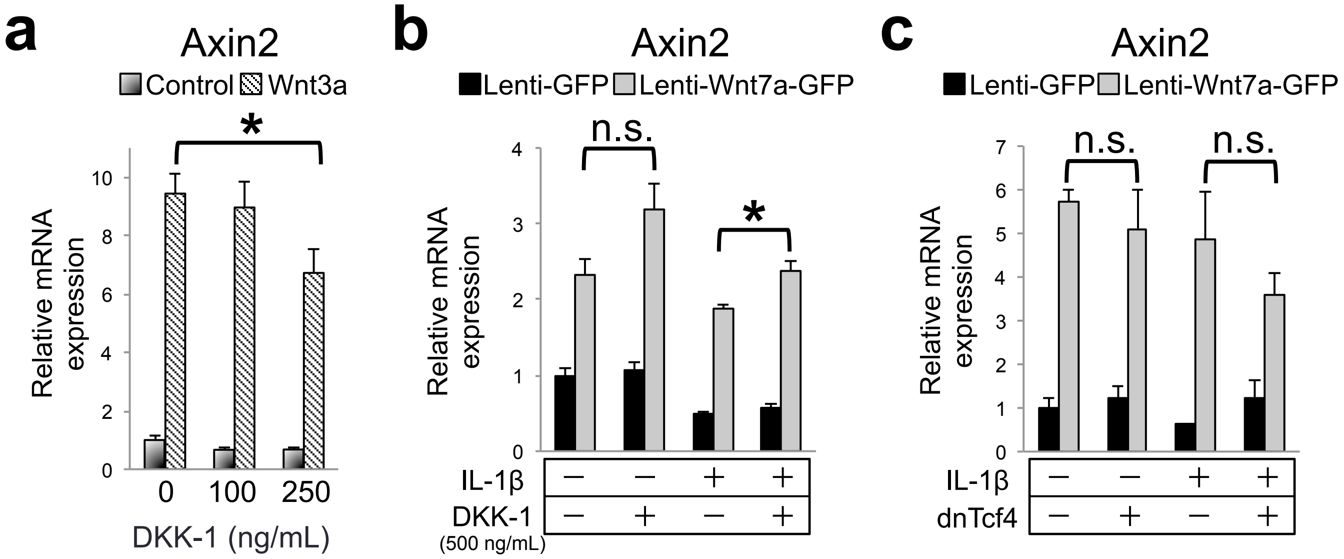

Supplementary Figure 2. DKK-1 reduces Wnt3a-induced Axin2 expression in human chondrocytes, but does not inhibit Wnt7a-induced Axin2 expression. a. RT-PCR analysis of Axin2 mRNA expression in nHACs cultured in Wnt3a or control conditioned media, in the presence of 0, 100, or 250 ng/mL DKK-1 for 2 days. Axin2 induction by Wnt3a was inhibited by DKK-1 in a dose-dependent manner. **b.** RT-PCR analysis of Axin2 mRNA expression in nHACs infected with lenti-GFP or lenti-Wnt7a-GFP with or without a higher level of DKK-1 (500 ng/mL) for 2 days. Axin2 induction by Wnt7a was unaffected by DKK-1 when IL-1β was not added, and was even increased when IL-1β (5ng/mL) was added. **c.** RT-PCR analysis of Axin2 mRNA expression in nHACs infected with lenti-GFP or lenti-Wnt7a-GFP, with or without dominant negative Tcf4 lentivirus (lenti-dnTcf4), and cultured with or without 5 ng/mL IL-1β for 2 days. Lent-dnTcf4 also did not significantly affect the Wnt7a induction of Axin2 mRNA expression. Analysis of variance (ANOVA) with post-hoc tests was used for evaluating the statistical significance between the gene expression of control vs. Wnt3a treated cells and lenti-GFP vs. lenti-Wnt7a treated cells across all of experimental conditions. All data are shown as mean ± SEM. * = $p < 0.05$.n.s. = not significant.

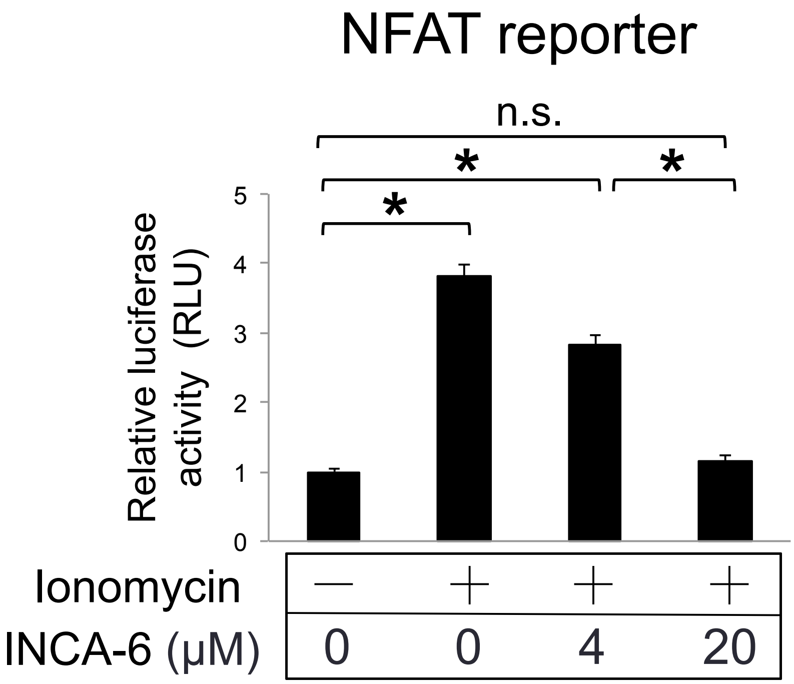

Supplementary Figure 3. The NFAT signaling inhibitor INCA-6 inhibits NFAT activity. NFAT luciferase reporter assay at 16 hours after treatment with or without 2 μM of ionomycin and 0, 4, or 20 μM of INCA-6. INCA-6 significantly reduced ionomycin activation of NFAT luciferase activity in a dose dependent manner. Analysis of variance (ANOVA) with post-hoc tests was used for evaluating the statistical significance between all experimental groups. Data are shown as mean ± SEM. * = $p < 0.05$. n.s. = not significant.

a Original films for Figure 5b

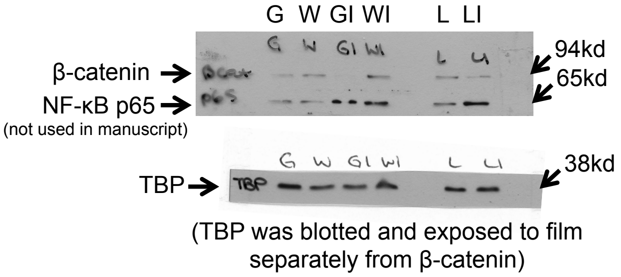

Legend
G = lenti-GFP
W = lenti-Wnt7a
GI = lenti-GFP + IL-1β
WI = lenti-Wnt7a + IL-1β
L = lithium chloride
(*control, not used in the manuscript*)
LI = lithium chloride + IL-1β
(*control, not used in the manuscript*)

b Original films for Figure 6a

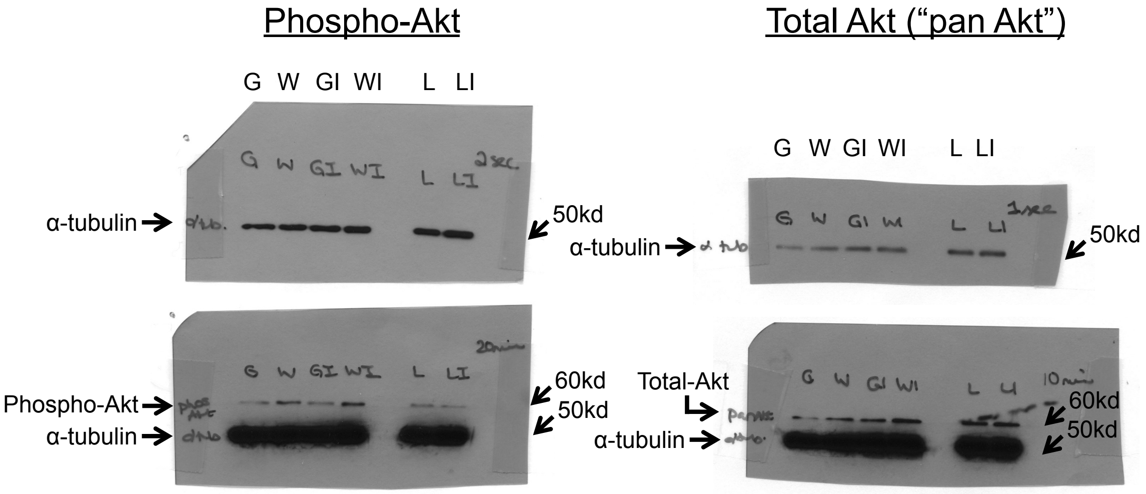

c Original films for Figure 7b (multiple exposures)

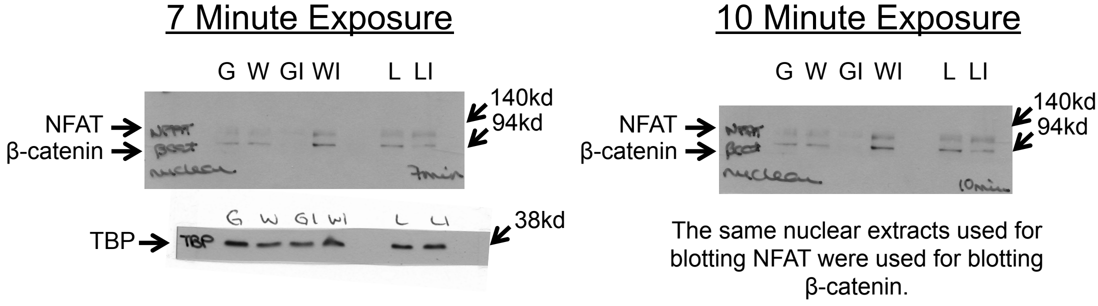

Original Western blot films for figures 5 - 7.
